# The Financial Presentation of Alzheimer’s Disease and Related Dementias

**DOI:** 10.1093/geroni/igaa057.2394

**Published:** 2020-12-16

**Authors:** Lauren Nicholas

**Affiliations:** Johns Hopkins University, Baltimore, United States

## Abstract

Dementia, a currently incurable degenerative cognitive disease, represents a major threat to financial stability. Early signs of dementia can include difficulties managing money and forgetting to pay bills, raising concerns about the implications of pre-clinical disease for financial well-being. We linked Medicare claims data to 20 years of consumer credit data for more than 80,000 older Americans living in single households to study the financial presentation of Alzheimer’s Disease and Related Dementias. Using non-parametric regression models, we find elevated rates of payment delinquency, subprime credit, and withdrawal from use of credit products up to 6 years before dementia is clinically diagnosed. Similar patterns did not appear with a number of placebo acute and chronic health conditions, suggesting that the adverse financial events are unique to dementia and do not occur with other acute or chronic illnesses. Part of a symposium sponsored by the Economics of Aging Interest Group.

